# Supplementary material for: Quantification of pollen viability in Lantana camara by digital holographic microscopy
Source: Quant Plant Biol. 2023 Jul 17;4:e7. doi: 10.1017/qpb.2023.5 (PMC10388712; doi:10.1017/qpb.2023.5)
Supplement: Supplementary file 1 [file S263288282300005Xsup001.docx]

**Quantification of Pollen Viability in *Lantana Camara* using Digital Holographic Microscopy**

*V. Kumar, N. Goyal, A. Prasad, S. Babu, K. Khare and G. Yadav*

Supplementary Material

This supplementary material provides additional information that is not part of the main manuscript but can be valuable for readers of our paper.

**1. Information provided by quantitative phase imaging vs. traditional phase contrast imaging**

The digital holographic microscopy (DHM) technology employed in this article is an emerging methodology that is not yet widely popular among plant Biology researchers. The readers may however be more familiar with the phase contrast modality nominally available in most research microscopes. As explained in Eq. (2) of our manuscript, when a spatially coherent beam of light is passed through a semi-transparent sample like pollen grains, the transmitted wavefront accumulates a phase distortion due to the position-dependent optical path difference experienced by the beam of light owing to the thickness and the (relative) refractive index distribution of the sample. Well-known theoretical description of the DHM and the phase contrast methodologies suggest that the DHM is able to extract this exact quantitative phase information by means of interference record. On the other hand, the traditional phase contrast methodology produces an enhanced intensity image where the image brightness distribution (to a first order in weak phase approximation) is directly related to the phase information. While DHM can provide quantitative phase information via phase reconstruction algorithms, the phase contrast filter can only provide qualitative visual information.


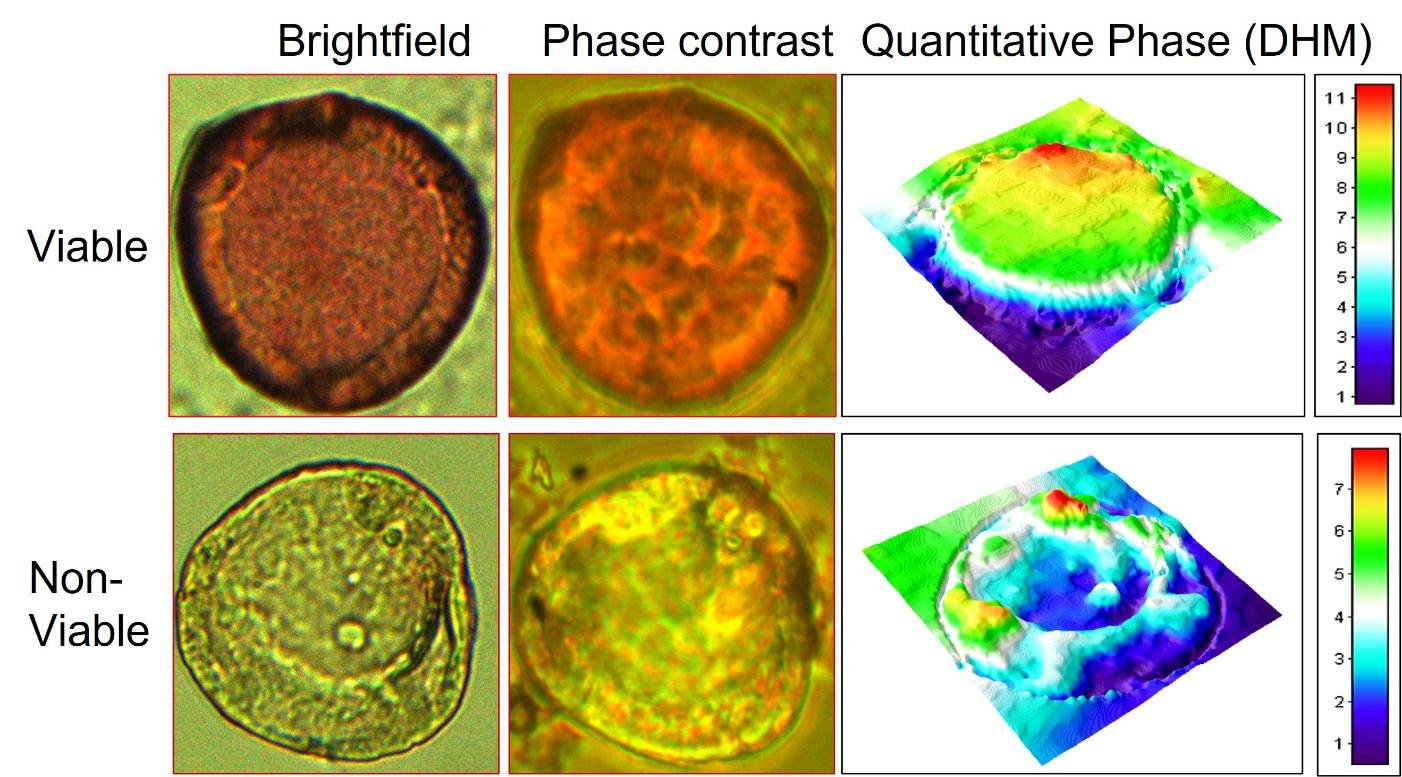


Fig. S1: Bright-field, phase contrast and quantitative phase images of a viable and a non-viable pollen grain all recorded in 40X microscopy configuration.

In Fig. S1, we provide an illustration of imaging of two pollen grains using bright-field, phase contrast and DHM based quantitative phase imaging for the benefit of readers. The samples were prepared using the same protocol described in Section 3.1.1 of the main manuscript. The bright-field and quantitative phase images were recorded using our dual mode DHM system (see Fig. 2 of the main manuscript). On the other hand, the phase contrast images of the same pollen were recorded using a separate traditional phase contrast microscope (make: Radical Scientific, model: RXLr-5RX). The same 40X magnification has been used to record the images. While phase contrast images appear to enhance or highlight some small features within the pollen, they do not readily provide any quantitative features that can be used to conclusively distinguish the two types of pollen grains. The phase images from DHM can however clearly illustrate distinct morphological features (as in Fig. 3 of the main paper) that may be interpreted as per Eq. (2) of our manuscript.
